# Crimean-Congo Hemorrhagic Fever Virus Kinetics in Serum, Saliva, and Urine, Iran, 2018

**DOI:** 10.3201/eid3008.240036

**Published:** 2024-08

**Authors:** Malihe Metanat, Seyed Dawood Mousavi Nasab, Tahmineh Jalali, Fahimeh Bagheri Amiri, Neda Sadat Torab Jahromi, Mahsa Tavakoli, Mohammad Hassan Pouriayevali, Mohammad Mehdi Gouya, Mostafa Salehi-Vaziri

**Affiliations:** University of Medical Sciences, Zahedan, Iran (M. Metanat, N.S. Torab Jahromi);; Pasteur Institute of Iran, Tehran, Iran (S.D. Mousavi Nasab, T. Jalali, F. Bagheri Amiri, M. Tavakoli, M.H. Pouriayevali, M. Salehi-Vaziri);; Iran University of Medical Sciences, Tehran (M.M. Gouya)

**Keywords:** Crimean-Congo hemorrhagic fever, vector-borne infections, arboviruses, zoonoses, viral kinetics, viruses, viral hemorrhagic fevers, laboratory diagnosis, emerging infectious diseases, Iran

## Abstract

Little is known about using noninvasive samples for diagnosing Crimean-Congo hemorrhagic fever (CCHF). We investigated detection of CCHF virus in serum, saliva, and urine samples. Our results indicate that serum is the best sample type for CCHF diagnosis; saliva can be used for noninvasive sampling.

Crimean-Congo hemorrhagic fever (CCHF), caused by CCHV virus (CCHFV), is an emerging disease that has been listed by the World Health Organization as a priority pathogen (*1*). Including noninvasive samples such as saliva and urine in testing can improve the current CCHF diagnostic algorithm. The World Health Organization has emphasized investigating the usefulness of alternative, noninvasive sample types, such as urine and oral fluid, for diagnosing and monitoring CCHF. For this study, we analyzed the utility of saliva and urine samples for laboratory diagnosis of CCHF by comparing real-time reverse transcription PCR (rRT-PCR) results for serially collected saliva, urine, and serum samples from CCHF patients in Iran. To provide additional information about the kinetics of CCHFV, we investigated the presence of RNA and antigen in saliva, urine, and serum samples, and IgM in serum samples. Ethics committees of the Zahedan University of Medical Sciences approved this study (IR.zaums.REC.1397.425). 

## The Study

We collected serum, saliva, and urine samples from 22 CCHF patients in Iran in 2018. Fourteen (63.6%) patients were male; mean age was 30.59 (SD = 12.83) years. We extracted viral RNA by using the QIAGEN QIAamp Viral RNA Mini Kit (https://www.qiagen.com) and the Fast-Track Diagnostics CCHF rRT-PCR (Siemens, https://www.siemens.com). We detected CCHFV IgM using a Vector-Best VectoCrimean-CCHF IgM Kit and antigens using a Vector-Best CCHFV-antigen ELISA Kit (https://en.vector-best.ru). We performed all experiments in a Biosafety Level 2-plus laboratory. We used GraphPad PRISM software (https://www.graphpad.com) for data analysis. 

**Figure 1:**
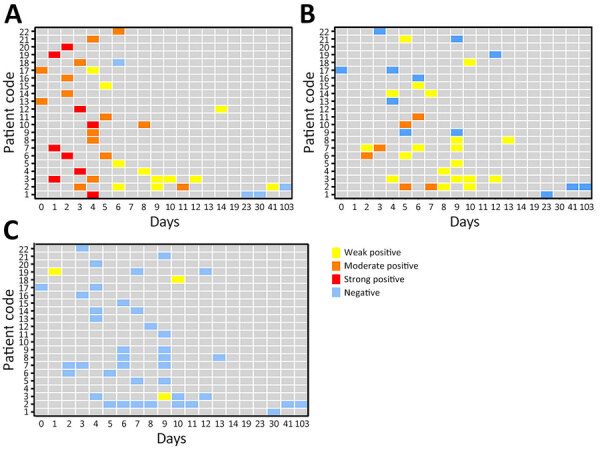
Results of Crimean-Congo hemorrhagic fever virus real-time reverse transcription PCR testing for serum (A), saliva (B), and urine (C) samples from 22 Crimean-Congo hemorrhagic fever patients in Iran, by days after onset of symptoms. Weak positive, cycle threshold (Ct) 31–36; moderate positive, Ct 21–30; strong positive, Ct ≤20.

We subjected serum, saliva, and urine samples from the 22 CCHF patients to rRT-PCR to identify CCHFV RNA, antigen ELISA for antigens, and IgM ELISA for IgM. We tested 39 serum, 37 saliva, and 42 urine samples using rRT-PCR and detected CCHFV RNA in all 3 types of samples but with different detection rates and time frames. Overall, 35/39 (89.74%) serum samples tested positive. We detected CCHFV RNA in 2 serum samples as early as day 0 and 1 serum sample as late as day 41. Overall, 25/37 (67.57%) saliva samples tested positive. We detected CCHFV RNA as early as day 2 in 2 saliva samples and as late as day 13 in 1 saliva sample. Only 3/42 (7.14%) urine samples tested positive (Figure 1). We detected viral RNA as early as day 1 in 1 urine sample and as late as day 10 in 1 urine sample. 

For further analysis, we categorized positive samples by days after symptom onset into 4 groups: 0–4, 5–9, 10–14, and ≥15 days (Table). In serum samples, the rRT-PCR positivity rate was high through day 14: 100% on days 0–4, 90.9% on days 5–9, and 100% on days 10–14. The positivity rate decreased substantially thereafter to 25% during days ≥15. rRT-PCR positivity rate for saliva samples gradually increased from 55.6% on days 0–4 to 83.3% on days 10–14; we observed no positive results during days ≥15. Rate of positivity in urine samples was very low during the first 2 weeks of the disease, and we observed no positive samples during days ≥15. 

**Table:** Positivity rate for Crimean-Congo hemorrhagic fever virus by real-time reverse transcription PCR and ELISA in serum, saliva, and urine samples from patients in Iran

Days after disease onset	PCR-positive samples (%)				Antigen ELISA–positive samples (%)			IgM ELISA–positive serum samples (%)
	Serum	Saliva	Urine		Serum	Saliva	Urine	
0–4	20/20 (100)	5/9 (55.6)	1/12 (8.3)		7/7 (100)	0/9 (0)	0/11 (0)	6/18 (33.3)
5–9	10/11 (90.9)	15/19 (78.9)	1/20 (5)		1/6 (16.7)	2/20 (10)	0/21 (0)	12/13 (92.3)
10–14	4/4 (100)	5/6 (83.3)	1/7 (14.3)		0/4 (0)	0/7 (0)	0/7 (0)	4/4 (100)
≥15	1/4 (25)	0/3 (0)	0/3 (0)		0/4 (0)	0/3 (0)	0/3 (0)	Not tested

**Figure 2:**
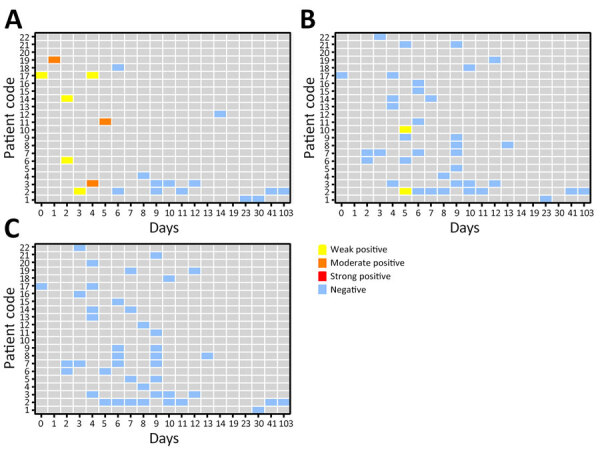
Results of Crimean-Congo hemorrhagic fever virus antigen ELISA testing for serum (A), saliva (B), and urine (C) samples from 22 Crimean-Congo hemorrhagic fever patients in Iran, by days after onset of symptoms. Weak positive, optical density (OD) <1; moderate positive, OD 1–3; strong positive, OD >3.

We tested 21 serum, 39 saliva, and 42 urine samples using Vector-Best CCHFV-antigen ELISA. Eight (38.10%) of 21 serum, 2/39 (5.13%) saliva, and 0/42 (0.0%) urine samples tested positive for CCHFV antigen (Figure 2). We detected CCHFV antigen as early as day 0 in 1 serum sample and as late as day 5 in 1 serum sample. In contrast, we detected CCHFV antigen in only 2 saliva samples on days 5–9, and no urine samples tested positive for CCHFV antigen. Antigen ELISA results were 100% positive in serum samples through day 5 of disease onset but declined substantially after that (Table). 

**Figure 3:**
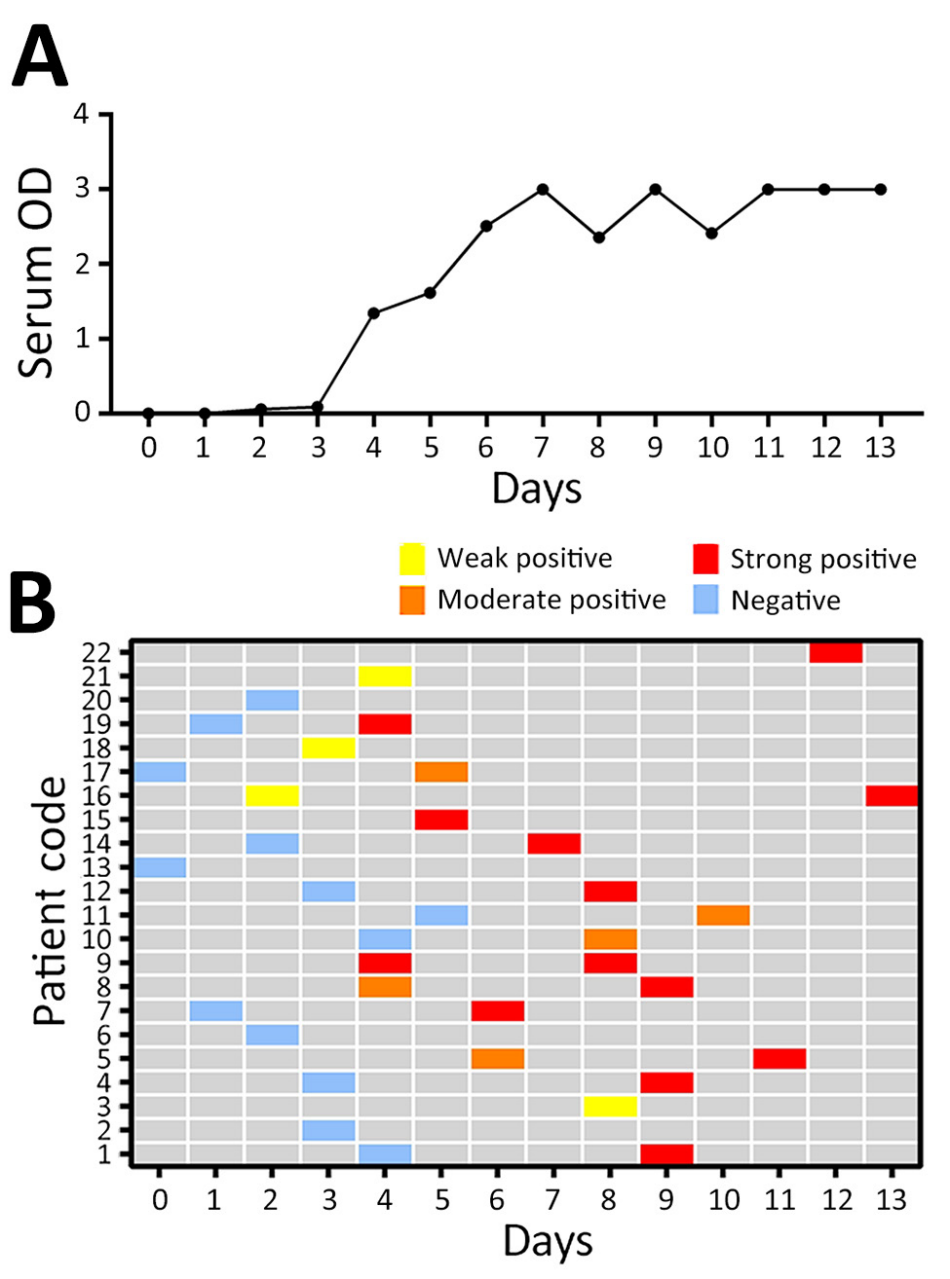
Results of Crimean-Congo hemorrhagic fever virus IgM antigen ELISA testing for serum samples from 22 Crimean-Congo hemorrhagic fever patients in Iran, by days after disease onset. A) Mean of detected OD in serum samples per day. B) IgM ODs. Weak positive, optical density (OD) <1; moderate positive, OD 1–3; strong positive, OD >3.

We analyzed 35 serum samples using a Vector-Best VectoCrimean-CCHF IgM kit. Of those serum samples, 22 (62.85%) were positive. On day 2, 1 serum sample tested positive for CCHFV IgM; by day 6, all 22 serum samples had tested positive for CCHFV IgM (Figure 3). The IgM positivity rate using CCHFV-IgM ELISA increased substantially during days 5–9 and reached 100% by days 10–14. We tested no samples for IgM during days ≥15 (Table). 

Our results showed that serum provides most suitable sample type for identifying CCHFV RNA. From highest to lowest, rRT-PCR positivity rates were 89.74% in serum, 67.57% in saliva, and 7.14% in urine. In line with our results, 1 study (*2*) identified CCHFV RNA in blood, saliva, and urine samples; highest positivity was observed in blood (100%, n = 8/8), followed by saliva (83%, n = 5/6) and urine (66%, n = 2/3). Similarly, another study (*3*), from Kosovo, identified CCHFV RNA in 66.66% of serum and 42.85% of urine samples from CCHF patients. In a study from India (*4*), viral RNA was detected in blood and urine samples, but the viral load in urine was lower. In a study conducted in Spain (*5*), viral RNA was detected in different sample types, including serum, saliva, and vaginal secretions, but not in urine. In our study, we detected viral RNA on different days after the onset of disease in all 3 evaluated sample types, but the widest diagnostic window (days 0–41) was related to serum samples. The study from Spain (*5*) also showed that CCHFV RNA can be detected in plasma samples through day 20 after onset of the disease. As in our review, the study from Kosovo (*3*) reported the lengthy persistence (36 days) of viral RNA in serum. 

We detected CCHFV antigen in serum and saliva but not urine samples. Although viral antigen was detectable in serum and urine samples, the positivity rate was much lower compared with the rate when tested for viral RNA. Only 38.1% of serum samples and 5.13% of saliva samples were positive for CCHFV antigen, supporting results from other studies suggesting the lower sensitivity of antigen ELISA compared with rRT-PCR testing (*6*,*7*). 

The genetic diversity of CCHF viruses can negatively affect the results of rRT-PCR (*8*). Therefore, targeting IgM along with viral RNA can be helpful to increase the sensitivity of the diagnosis. In our study, IgM was detectable in serum from the second day of the disease and, from day 6 onward, all serum samples were positive for IgM. Of note, CCHFV antibodies cannot be detected in fulminant CCHF patients (*9*,*10*). Therefore, interpretation of serologic tests should be considered in the context of the patient’s clinical and laboratory data. 

## Conclusion

This study showed that CCHF virus can be identified in serum, saliva, and urine samples. However, for laboratory diagnosis of acute CCHFV infection, the best sample type was serum and the best target was viral RNA. Between saliva and urine as noninvasive samples, saliva might be the more suitable option for genome identification. 
